# A rare case of neonatal colonic obstruction caused by a solitary intestinal tumor

**DOI:** 10.1186/s40792-021-01107-9

**Published:** 2021-01-19

**Authors:** Yu Sugai, Yutaka Hirayama, Yasushi Iinuma, Kengo Nakaya, Takato Aikou, Shotaro Taki, Hideki Hashidate, Yoshiaki Kinoshita

**Affiliations:** 1 Department of Pediatric Surgery, Niigata-City General Hospital, 463-7 Shumoku, Chuo-ku, Niigata City, Niigata, 950-1197 Japan; 2 Department of Pediatric Surgery, Nagaoka Red Cross Hospital, 2-297-1 Senshu, Nagaoka City, Niigata, 940-2085 Japan; 3 Department of Pathology, Niigata-City General Hospital, 463-7 Shumoku, Chuo-ku, Niigata City, Niigata, 950-1197 Japan; 4 Division of Pediatric Surgery, Niigata University Graduate School of Medical and Dental Sciences, 1-757 Asahimachi-dori, Chuo-ku, Niigata City, Niigata, 951-8510 Japan

**Keywords:** Neonatal tumor, Intestinal obstruction, Napkin ring, Myofibroma

## Abstract

**Background:**

Intestinal obstruction caused by a tumor is very rare in newborns, and the preoperative diagnosis is difficult. We herein report a rare case of neonatal colonic obstruction due to solitary intestinal myofibroma with characteristic findings on gastrografin enema and the surgical strategy.

**Case presentation:**

A 4-day-old female infant presented to our neonatal intensive-care unit with abdominal distention and bilious vomiting after feeding. A gastrografin enema showed that the transverse colon near the hepatic flexure was not delineated at the oral side. When pressure was applied, a small amount of contrast material moved into the mouth in the form of threads. Microcolon was not observed, and stenosis of the transverse colon was found 9 cm from the Bauhin valve. Partial resection and end-to-end anastomosis were performed. A pathological examination of the resected specimen suggested gastrointestinal stromal tumor (GIST). After obtaining a second opinion, the histology and immunohistological markers were deemed characteristic of infantile myofibroma.

**Conclusion:**

If string sign and a napkin ring appearance are found in a case of neonatal intestinal obstruction, surgery should be performed with a tumor in mind. In cases of neonatal intestinal obstruction caused by a tumor, the lesion should be resected with a sufficient surgical margin before the pathological examination.

## Background

Neonatal intestinal obstruction may be due to a variety of conditions, including atresia and stenosis, annular pancreas, malrotation, duplication cyst, meconium ileus, meconium plug syndrome and neonatal small left colon syndrome, Hirchsprung’s disease, tumor, trauma, and other rarer causes [1]. However, intestinal obstruction caused by a tumor is very rare in newborns and difficult to diagnose preoperatively.

Infantile myofibromatosis, although a rare disorder, is a common tumor of infancy and childhood [2]. The disease can be solitary or generalized [3]. It is manifested by the formation of tumors in the skin, muscle, viscera, bone, and subcutaneous tissues [4]. Visceral involvement is found in only 19% of cases [5]. Solitary intestinal myofibroma (SIM) is rare and solitary colonic involvement is extremely rare. A typical presentation of SIM involves intestinal perforation or obstruction [6].

We herein report a rare case of neonatal colonic obstruction due to SIM with characteristic findings on gastrografin enema and the surgical strategy.

## Case presentation

**Fig. 1:**
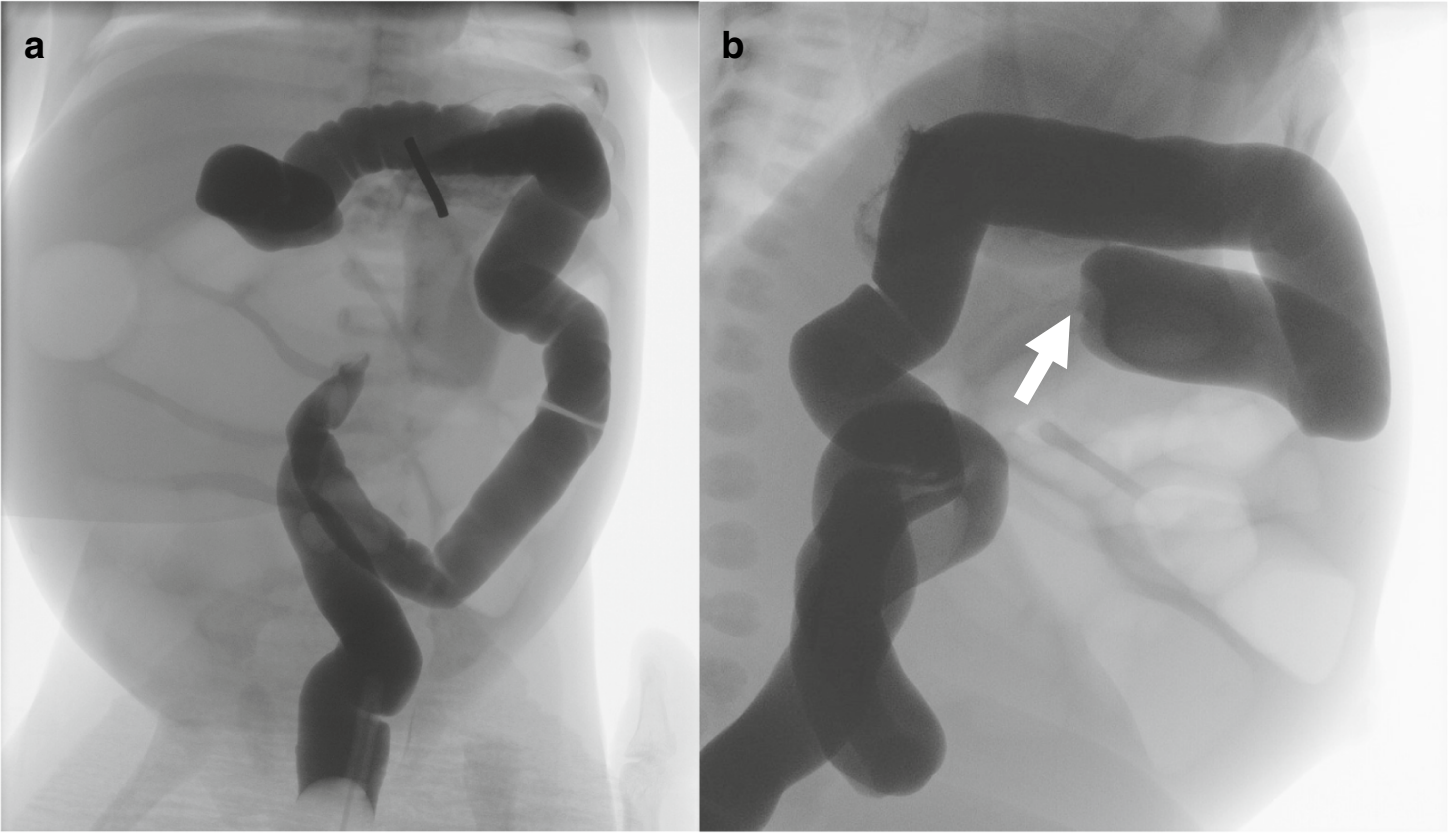
Preoperative gastrografin enema. **a** Frontal view. The transverse colon near the hepatic flexure was not delineated on the oral side. **b** Lateral view. The string sign was observed at transvers colon (white arrow)

The present patient was a 4-day-old female infant. The mother had had a natural pregnancy. The patient had been born by vaginal delivery at a gestational age of 39 weeks 3 days with a birthweight of 3124 g. The passage of meconium was observed soon after the birth. The patient presented on day 4 of age to our neonatal intensive-care unit with abdominal distention and bilious vomiting after each feed. Her vital signs were stable, and she was slightly inactive and mildly dehydrated. Upper gastrointestinal series showed a normal appearance. Gastrografin enema showed that the transverse colon near the hepatic flexure was not delineated on the oral side. When pressure was applied, the string sign was observed (Fig. 1). Microcolon was not found. Shortly after admission, exploratory laparotomy was performed under suspicion of intestinal stenosis caused by intussusception or volvulus.

**Fig. 2:**
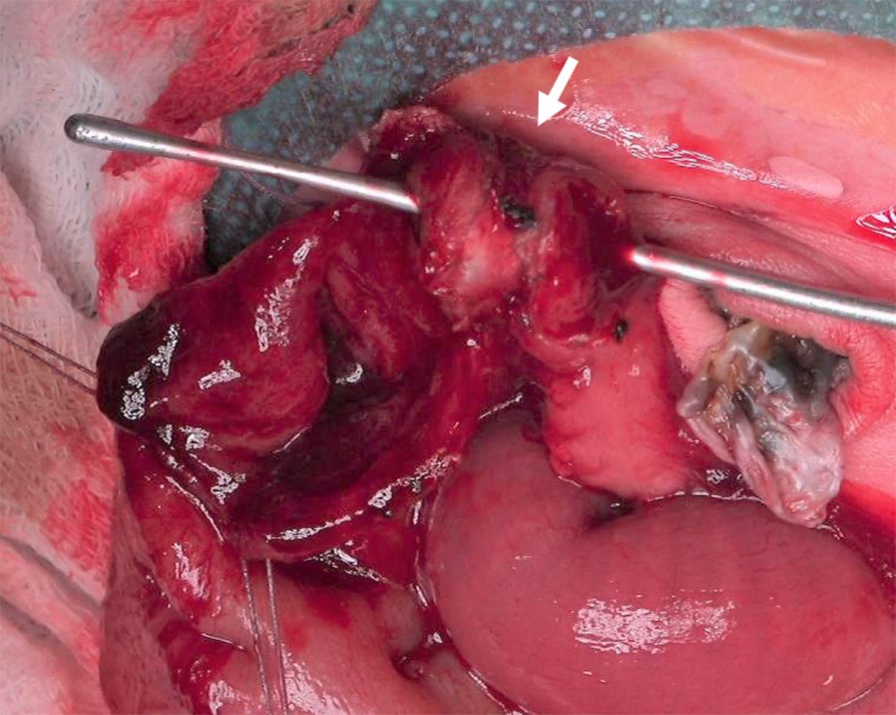
Macroscopic findings from surgery. Intraoperative findings were the napkin ring appearance at transvers colon (white arrow). A bowel incision was made in the vicinity of the stenosis to see if a sonde could pass through the stenosis. A partial resection and end-to-end anastomosis were performed

Stenosis of the transverse colon was found 9 cm from the Bauhin valve. It was elastically hard, white, totally fibrotic, and had a ‘napkin ring’ appearance [7]. The sonde passed through the stricture. We suspected organizing due to intussusception in the prenatal period. Partial resection and end-to-end anastomosis were thus performed (Fig. 2).

**Fig. 3:**
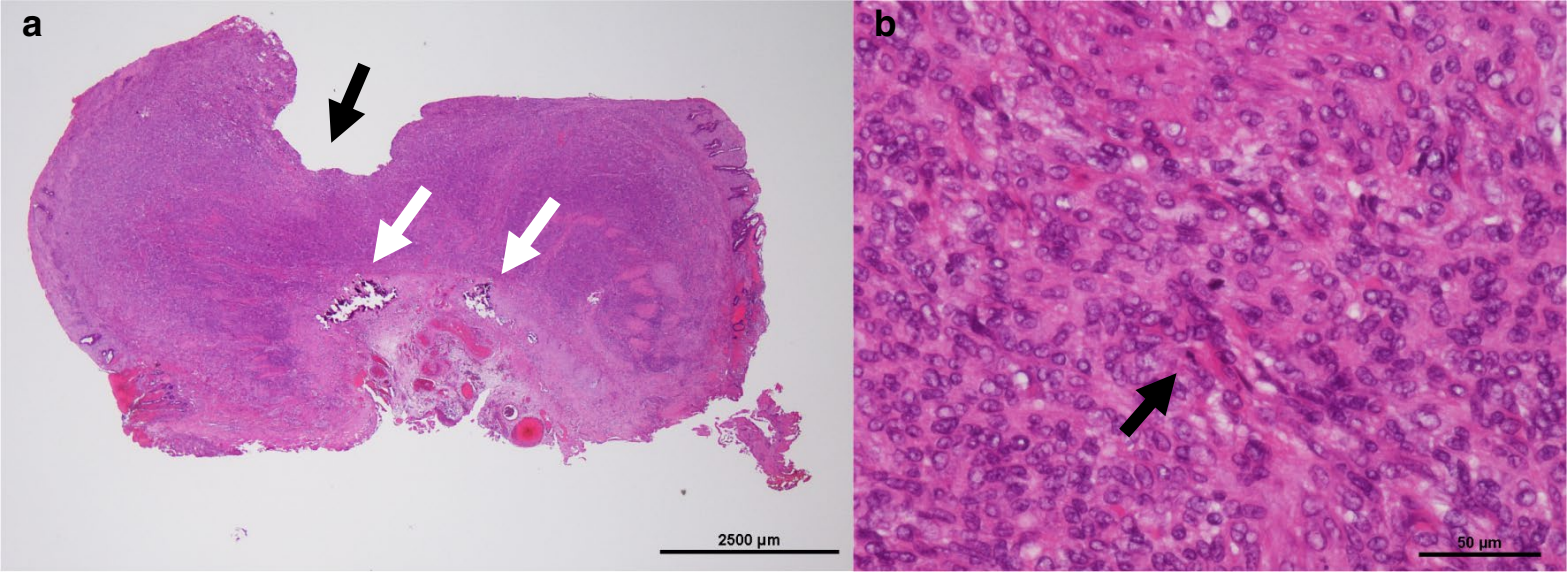
Hematoxylin and eosin staining. **a** Tumor cells were proliferating in the submucosal to muscular layers in a generalized manner. The mucosa was also infiltrated by tumor cells and the luminal surface was ulcerated (black arrow). There was partial calcification near the serous layer (white arrow). **b** Strongly enlarged and multiple spindle-shaped cells were observed (black arrow)

A pathological examination of the resected specimen showed that the cells were mainly composed of spindle-shaped cells, with some round cells and slightly clear cells mixed in. They were circumferential, proliferating from the submucosa to within the muscle layer and narrowing. The mucosal surface was ulcerated. The mucous membrane was atrophied and atrophic. Calcification was observed in the muscle layer. There was no obvious epithelioid pattern (Fig. 3). An immunohistochemical analysis revealed positive staining for vimentin, cluster of differentiation 34 (CD34), and discovered on GIST-1 (DOG1), weak positive staining for c-kit, and negative staining for cytokeratin AE1/AE3, small muscle actin (SMA), and S-100 protein (S-100). The Ki-67 index was 30%, and the mitotic index was 115/50 high-power field (HPF).

Given the above, we suspected a malignant tumor of gastrointestinal stromal tumor (GIST). A further consideration, including an immunohistochemical analysis revealed positive staining for SMA, CD34, DOG-1, and SDHB and negative staining for c-kit, S-100, β-catenin, pan-tropomyosin resepter kinase (pan-TRK), analplastic lymphoma kinase (ALK), and c-ros oncogene 1 (ROS1). The histology and immunohistological markers were characteristic of infantile myofibroma.

The patient had an uncomplicated postoperative course and was discharged home after 3 weeks. Whole-body magnetic resonance imaging (MRI) and gastrografin enema performed 6 months after the operation showed no recurrence of the lesion and no other new lesions.

## Discussion

Neonatal intestinal obstruction occurs 1 in 1500 live births [8]. Intestinal atresia is the most common cause of neonatal intestinal obstruction [9]. Neonatal intestinal obstruction due to a tumor is very rare. SIM was first described by Kauffma and Sout in 1965. Twenty-one cases have been described since 1965. Fifteen of these were in neonates, 14 of which presented with intestinal obstruction or perforation [6]. Our case is the fifth one showing SIM in the colon, and the fourth involving a neonate (Table 1). No fetal abnormalities were noted in any of the colon cases. Most of the colon cases occurred during the neonatal period and all cases had a good prognosis. There have been no reports suggesting the timing of SIM occurrence. In our case, fetal echocardiography did not indicate excessive amniotic fluid, the passage of meconium had been observed soon after birth, and calcification was observed within the tumor. Therefore, we suspected the tumor had developed in the third trimester.

**Table 1:** Overeview of solitary intestinal myofibromas in the colon

Year	Authors [Ref.]	Age	Sex	Birth weight	Clinical presentation	Meconium	Location	Stenosis	Operation
1984	Srigley and Mancer [10]	2 days	M		Obstruction, prenatal perforation		Transverse	Localization	Resection, colostomy
1997	Al-Salem et al. [11]	4 days	F	3000 g	Intestinal perforation	+	Descending		Resection, colostomy
2000	Lacson et al. [12]	5 months	M		Intestinal obstruction	+	Splemoc flexure	Circumferential	Resection, anastomosis
2002	Numanoglu et al. [13]	6 days	M	3600 g	Intestinal obstruction	+	Transverse	Circumferential	Resection, colostomy
2020	Present case	4 days	F	3124 g	Intestinal obstruction	+	Transverse	Circumferential	Resection, anastomosis

Our case showed characteristic findings on gastrografin enema. Although the preoperative findings were atypical for intussusception, emergency surgery was performed. The intraoperative findings showed a napkin ring appearance. A “napkin ring” appearance is used to describe the morphology of the gastrointestinal tract in cases of circumferential stenosis forming a ring [7], and it is commonly observed on X-ray of circumferential stenosis of small intestinal cancers and colonic ganglia tumors [7, 14]. In the present case of SIM, the surgical specimen was described as a “napkin ring”, and the barium enema findings were described as an “apple core”. In 11 of 21 SIM cases, the surgical specimen showed circumferential stenosis. We, therefore, believe that the string sign on gastrografin enema and a “napkin ring” appearance of the specimen are common findings of neonatal intestinal obstruction due to SIM. It is also important to check for the presence of microcolon to differentiate it by congenital colonic stenosis.

Initially, GIST was suspected on the pathological examination of this case. Additional resection was, therefore, considered. After obtaining a further consideration, including an immunohistochemical analysis, the final diagnosis of SIM was made. Additional resection is highly invasive in all patients, not just neonates, but the risk of short bowel syndrome is increased in neonates compared with older patients. In a single-center review, the ratio of benign to malignant pediatric gastrointestinal tumors was 9:4 [15]. In general, tumor resection helps improve the prognosis of pediatric tumors, regardless of whether they are benign or malignant. In our case, a tumor was not included in the differential diagnosis at the time of surgery, although it should have been, based on the enema findings. If a tumor is suspected, it should be resected with a sufficient margin, regardless of whether it is benign or malignant.

The prognosis for solitary myofibroma cases is good with complete resection [6]. No reports have described metastasis or local recurrence after complete resection of SIM [11, 13, 16]. On the other hand, the symptoms of generalized myofibroma cases are site-specific and varied. Gastrointestinal lesions were found to be present in 35% of cases, with a mortality rate of 76% [4]. Therefore, SIM is necessary complete resection. Before or after resection, it is necessary to perform a whole-body search to confirm other lesions. MRI is particularly useful for such ends [6], and in the present case, MRI was performed 6 months after surgery, with no evidence of systemic lesions or recurrence found. In recent years, a genetic component has been suggested to exist in the familial form of infantile myofibromatosis, due to autosomal dominant inheritance and mutations in the PDGFRB gene [17, 18]. There was no family history in this case. However, the genetic details are unknown, as no genetic testing has been performed.

## Conclusion

We herein report a rare case of neonatal colonic obstruction caused by SIM. This is the first case in the world in which a “napkin ring” was confirmed by neonatal gastrografin enema. If neonatal intestinal obstruction includes enema findings of string sign and the “napkin ring” appearance, a tumor should be considered. In cases of neonatal intestinal obstruction caused by a tumor, the lesion should be resected with a sufficient surgical margin before the pathological examination.

## Data Availability

Not applicable.

## Abbreviations

SIM: Solitary intestinal myofibroma
GIST: Gastrointestinal stromal tumor
CD34: Cluster of differentiation 34Discovered
DOG2: Discovered on GIST-1 (DOG1)
SMA: Small muscle actin
S-100: S-100 protein
HPF: High-power fields
Pan-TRK: Pan-tropomyosin resepter kinase
ALK: Analplastic lymphoma kinase
ROS1: C-ros oncogene 1
MRI: Magnetic Resonance Imaging
